# The Role of Radiotherapy, Chemotherapy, and Targeted Therapies in Adult Intramedullary Spinal Cord Tumors

**DOI:** 10.3390/cancers16162781

**Published:** 2024-08-06

**Authors:** Ines Esparragosa Vazquez, François Ducray

**Affiliations:** Neuro-Oncology Department, Hospices Civils of Lyon, 69500 Bron, France; ines.esparragosa-vazquez01@chu-lyon.fr

**Keywords:** spinal cord tumor, intramedullary tumors, spinal ependymomas, spinal astrocytomas, neuro-glial tumors, hemangioblastomas

## Abstract

**Simple Summary:**

Intramedullary primary spinal cord tumors represent a heterogeneous group of very rare tumors. In cases of recurrence or high-grade tumors, radiotherapy, chemotherapy, or targeted therapies can be indicated. The purpose of this review is to clarify the actual knowledge about intramedullary primary spinal cord tumors in light of the latest progress made in their classification.

**Abstract:**

Intramedullary primary spinal cord tumors are rare in adults and their classification has recently evolved. Their treatment most frequently relies on maximal safe surgical resection. Herein, we review, in light of the WHO 2021 classification of central nervous system tumors, the knowledge regarding the role of radiotherapy and systemic treatments in spinal ependymomas, spinal astrocytomas (pilocytic astrocytoma, diffuse astrocytoma, spinal glioblastoma IDH wildtype, diffuse midline glioma H3-K27M altered, and high-grade astrocytoma with piloid features), neuro-glial tumors (ganglioglioma and diffuse leptomeningeal glioneuronal tumor), and hemangioblastomas. In spinal ependymomas, radiotherapy is recommended for incompletely resected grade 2 tumors, grade 3 tumors, and recurrent tumors not amenable to re-surgery. Chemotherapy is used in recurrent cases. In spinal astrocytomas, radiotherapy is recommended for incompletely resected grade 2 astrocytomas and grade 3 or 4 tumors as well as recurrent tumors. Chemotherapy is indicated for newly diagnosed high-grade astrocytomas and recurrent cases. In hemangioblastomas not amenable to surgery, radiotherapy is an effective alternative option. Targeted therapies are playing an increasingly important role in the management of some intramedullary primary spinal cord tumor subtypes. BRAF and/or MEK inhibitors have demonstrated efficacy in pilocytic astrocytomas and glioneuronal tumors, belzutifan in von Hippel–Lindau-related hemangioblastomas, and promising results have been reported with ONC201 in diffuse midline glioma H3-K27M altered.

## 1. Introduction 

Intramedullary primary spinal cord tumors are rare tumors that represent less than 5% of central nervous system (CNS) tumors [1]. They are more frequent in children than in adults [2,3,4,5]. In adults, intramedullary primary spinal cord tumors most frequently consist of ependymomas (60–70%), astrocytomas (30–40%), neuro-glial tumors, and hemangioblastomas [1,5,6]. Their treatment primarily relies on surgical resection, which may be curative for well-circumscribed lesions. Nevertheless, for incompletely resected, surgically unresectable tumors, high-grade tumors or recurrent tumors radiation therapy (RT) and systemic treatment can be indicated. Given the rarity of intramedullary spinal cord tumors, most of the evidence regarding the role of these treatments relies on retrospective series. The definition of the optimal postoperative management of intramedullary spinal cord tumors is also complicated by the fact that their classification has significantly changed within the last 15 years (Table 1). Herein, we review, in light of the WHO 2021 classification of central nervous system tumors, the current knowledge regarding the role of RT and systemic treatments in adult spinal ependymomas, spinal astrocytomas (including pilocytic astrocytomas, diffuse astrocytomas, spinal glioblastomas, diffuse midline gliomas H3 K27M altered, and high-grade astrocytomas with piloid features), glioneuronal tumors (i.e., gangliogliomas and diffuse leptomeningeal glioneuronal tumors), and hemangioblastomas (Table 2). 

## 2. Spinal Ependymomas

### 2.1. Spinal Intramedullary Ependymomas

Spinal ependymomas are slightly more frequent in males than in females and typically occur during the 3rd and 6th decades [4,7]. In the WHO 2021 classification, ependymomas are classified according to their location and histopathological and molecular features [8]. Ependymal tumors located in the spinal cord are currently classified as spinal subependymomas (grade 1), spinal ependymomas (grade 2 or grade 3), and myxopapillary ependymomas (grade 2) [8,9,10]. Although, at the histological level, spinal ependymomas share many similarities with their intracranial counterpart, their genetic and methylation profiles are different. The most common genetic alteration in spinal ependymomas consists of NF2 alterations (copy loss and/or mutations) [8]. Recently, a new variant of spinal cord ependymoma has been identified based on methylation profiling. This variant referred to as MYCN-amplified spinal cord ependymoma in the WHO 2021 classification is defined by the presence of a MYCN amplification and is considered as grade 3. It is characterized by an aggressive clinical presentation, frequent leptomeningeal dissemination, and resistance to treatments [8,9,11]. Spinal ependymomas are most frequently located in the cervical spine, except myxopapillary ependymomas that are located in the filum terminale and are not strictly speaking intramedullary spinal cord tumors [10,12]. Grade 2 and grade 3 ependymomas are characterized by a risk of local recurrence and distant leptomeningeal dissemination despite maximal resection and RT [13]. Therefore, it is important to evaluate the entire craniospinal axis with spinal and brain MRIs and to analyze the cerebrospinal fluid (CSF) at diagnosis and at the time of recurrence [10].

The primary treatment option for patients with spinal ependymomas and subependymomas is maximum safe resection. Complete resection is the most important prognostic factor [9,10]. In subependymomas and grade 2 spinal ependymomas, the risk of recurrence after complete resection is low, around 11 and 30% at 5 years depending on series [3,5,9,14,15]. In patients with grade 3 spinal ependymomas, gross total resection (GTR) has been shown to increase overall survival (OS) compared to subtotal resection (STR) or biopsy, but the risk of recurrence in these tumors remains important (around 30% after GTR and 55% after STR) [16].

Overall, spinal ependymomas are associated with prolonged progression-free survival (PFS) and OS with a median of 82 and 180 months, respectively [5]. Nevertheless, subtotally resected spinal ependymomas tend to recur at rates up to 50–70% without adjuvant therapy [14]. In recurrent ependymomas, re-surgery or re-irradiation should therefore be considered. In brain ependymomas, these treatments have been associated with an improved outcome [17]. In contrast, the role of chemotherapy in spinal ependymomas is less well established, and chemotherapy is generally considered only in patients in whom local treatments are not possible anymore (re-surgery or re-irradiation) [7]. Given their rarity, most studies on the role of chemotherapy in spinal ependymoma consist of retrospective studies with a small number of patients [12,13,18,19,20,21,22,23,24,25,26]. In addition, they were conducted before the WHO 2021 classification, and for example, there is no specific data regarding the treatment of the recently described MYCN-amplified spinal cord ependymoma entity. Therefore the recommendations that can be driven from these studies should be applied with caution after discussion in specialized multidisciplinary tumor boards.

Based on the available studies and despite their previously mentioned limitations, focal RT (45 to 54 Gy) is recommended for patients with grade 2 spinal ependymomas in whom complete resection is not possible [5,9,12,14]. This recommendation is based on several retrospective studies showing that RT improves PFS [15,27,28,29,30]. Postoperative RT, however, does not improve OS [7,16,31]. In patients in whom GTR is achieved, RT is not recommended [9].

Because of their high proliferative rate and great propensity for tumor infiltration, postoperative RT is recommended for grade 3 spinal ependymomas, even after GTR [5,7]. Focal RT (45–54 Gy) is indicated in patients without leptomeningeal dissemination and craniospinal irradiation in those with evidence of leptomeningeal dissemination [10,12]. In the case of craniospinal irradiation, the recommended dose is <36 Gy at 1.5–1.8 Gy/fraction to the entire craniospinal axis and a boost of 45–54 Gy to the primary tumor site [5,9,10,30]. As for grade 2 spinal ependymomas, postoperative RT has been shown to improve PFS but not OS in grade 3 spinal ependymomas [16].

In spinal ependymomas, chemotherapy is considered at the time of recurrence, when re-surgery or re-irradiation is not possible anymore. However, its role and efficacy remain unclear as most studies were retrospective and included both patients with intracranial and spinal ependymomas. Chemotherapy regimens used in recurrent spinal ependymomas include temozolomide (TMZ), TMZ combined with lapatinib, platin-based regimens, etoposide, and bevacizumab-based regimens [12,18,20,21,32,33,34,35]. There is currently no data to help clinicians in the choice of first-line chemotherapy. Nevertheless, the more robust data come from studies that assessed the efficacy of TMZ alone or in combination.

The efficacy of TMZ alone (or in combination with cisplatin) in recurrent spinal ependymomas has been assessed in a few retrospective studies [18,20,32]. In these studies, 36% to 50% of patients showed stable disease (SD) as the best response rate, with a median PFS of 2 to 10 months [18,20,32]. A single-arm phase 2 trial studied the efficacy of dose-dense TMZ in combination with lapatinib in patients with recurrent brain and spinal ependymomas [12]. The rationale for combining TMZ with lapatinib relied on the fact that this treatment targets the epidermal growth factor receptor (ErbB1) and the related family member human epidermal growth factor receptor 2/neu (ErbB2), which are both overexpressed on ependymoma tumor cells [36]. In this single-arm study, 50 patients received TMZ at a dose of 125 mg/m^2^ as a single daily dose on days 1–7 and 15–21 of a 28-day cycle in combination with a single daily dose of lapatinib 1250 mg orally. Treatment was generally well-tolerated. In the subgroup of patients with recurrent spinal ependymomas (*n* = 25), the median PFS was 0.9 years, and tumor response was observed in eight patients (32%) consisting of two complete responses (CRs) and six partial responses (PRs). In 8/25 patients (32%), a significant clinical improvement was observed [12].

The efficacy of carboplatin and cisplatin is less robust as they have been assessed in a few studies that included both recurrent intracranial and spinal ependymomas but mostly intracranial tumors [18,34,37]. In these studies, the response rate (including CR, PR, and SD) was 67% to 84.6% with a median 6-month PFS between 6 and 11 months. In two studies, the efficacy of a platin-based regimen seemed superior to that of nitrosoureas [18,34]. Unfortunately, the efficacy of platin-based regimens was not specifically provided for spinal ependymomas. In a small prospective study of 10 patients with recurrent low-grade spinal ependymomas, Chamberlain et al. assessed the efficacy of oral etoposide (50 mg/m^2^/day in a 3-week on-schedule and 2-week off-schedule) and reported a median PFS and OS of 15 and 17.5 months, respectively [19].

The best evidence regarding the efficacy of bevacizumab in spinal ependymomas comes from studies conducted in NF2 patients. Farschtschi et al. reported a response rate of 80% in a series of 8 patients with NF2-related spinal ependymomas with a median PFS of 12 months [38]. In another series, a clinical and radiological improvement was noted in 24/41 patients (59%) with NF2-related spinal cord ependymoma [21]. However, in NF2 patients, bevacizumab is usually given in treatment-naïve patients with unresectable ependymomas. The efficacy of bevacizumab in recurrent spinal ependymomas occurring after RT in non-NF2 patients is less clear, but retrospective series and case reports have reported potential benefit [13,21,23,35,39,40]. A small retrospective series on 8 patients reported a 75% response rate with a median PFS of 5 in patients with recurrent ependymomas. In this series, however, patients had mostly intracranial ependymomas, and bevacizumab was associated with other chemotherapy regimens [13].

Besides lapatinib, there is currently little role for targeted therapies in spinal ependymomas. Several clinical trials are evaluating the efficacy of brigatinib (an ALK inhibitor), neratinib (an EGFR and HER 2 inhibitor), or selumetinib (a MEK inhibitor) in patients with NF2-associated progressive tumors (NCT04374305, NCT03095248). Blackwood et al. reported the case of a patient diagnosed with an NF2-related spinal ependymoma treated by everolimus and selumetinib who showed a PR after 3 months of treatment [24]. Nevertheless, in a phase 2 study, everolimus demonstrated no efficacy in recurrent pediatric spinal grade 2 or 3 ependymomas [25]. Regarding immunotherapy, a phase 2 study is investigating the PD1 inhibitor nivolumab (NCT03173950) in adult patients with rare CNS tumors including spinal ependymomas. However, retrospective series of checkpoint inhibitors in pediatric patients with spinal ependymomas have been disappointing until now [41,42]. In the future, the high level of expression of HER2 in spinal ependymomas may constitute a therapeutic opportunity for the use of antibody–drug conjugates targeting HER2 [43]. In conclusion, there is an important need for prospective studies to clarify the role of both radiotherapy and chemotherapy in spinal intramedullary ependymomas.

### 2.2. Myxopapillary Ependymomas

Myxopapillary ependymomas represent a distinct form of spinal ependymomas. According to the 2021 WHO classification of CNS tumors, they are now considered as grade 2. They are typically located in the cauda equina and the conus medullaris. Although considered as grade 2, myxopapillary ependymomas frequently demonstrate leptomeningeal dissemination with a rate between 30% and 50% according to the series. Primary treatment relies on GTR. When GTR is not possible, especially when adhesion to nerve roots is too important, postoperative RT is indicated, which leads to acceptable disease control. This recommendation is based on retrospective studies that have shown that RT increased the 10-year PFS from 40% to 70% compared to patients treated with surgery alone [9,44]. In cases of recurrent disease, if local treatment is not possible (re-surgery or RT), retrospective series suggest that chemotherapy with TMZ can sometimes result in prolonged disease control [4,22]. As other options of systemic treatment, a case report has suggested the benefit of combining TMZ with the PARP inhibitor olaparib [26]; another case report described a prolonged SD in a patient with a metastatic myxopapillary ependymoma treated with the programmed death 1 (PD1) inhibitor tislelizumab [23], but data are limited and supplementary studies will be necessary to determine the therapeutic potential of these treatments.

## 3. Spinal Astrocytomas

Depending on the series, spinal cord astrocytomas represent 30–40% of intramedullary tumors in adults and 80–90% of intramedullary tumors in children. They are considered the most common pediatric spinal cord tumor [3,6,45]. The most common localization is the thoracic spine in adults and the cervical/cervicothoracic spine in children [2,46]. Due to their low frequency and the recent change in their histo-molecular classification, no prospective studies have been published to date, and treatment recommendations are most frequently based on small retrospective series or on what is known regarding their cerebral counterparts. Another limitation is that many retrospective studies on spinal astrocytomas included tumors that would now be considered as distinct entities according to the WHO 2021 classification. Although the optimal classification of spinal astrocytomas remains to be fully established, based on the WHO 2021 classification, they can currently be divided into the following entities: pilocytic astrocytoma (PA), diffuse astrocytoma (DA), spinal glioblastoma IDH wildtype (GBM), diffuse midline glioma H3 K27M altered (DMG-H3), and high-grade astrocytoma with piloid features (HGAP) [8,47,48].

### 3.1. Spinal Pilocytic Astrocytomas 

Pilocytic astrocytoma (PA) is a WHO grade 1 slow-growing circumscribed glioma [49,50]. In adults, it represents around 1.5% of CNS tumors. In adults, PA rarely (around 2% of PA) occurs in the spinal cord [50,51]. When they do, they are typically observed in patients around 30 years old [49]. The most common locations are the cervical and thoracic regions [50]. Spinal PAs harbor a KIAA1549–BRAF fusion in 60–70% of cases and a BRAF V600E mutation in 10% of the cases [49,52]. In the pediatric population, the presence of a KIAA1549–BRAF fusion has been associated with a better prognosis compared to that of a BRAF V600E mutation [49,52].

The standard treatment of spinal PA is surgical resection [50,53]. After GTR or STR, a watch-and-wait approach is the standard of care [49]. The largest retrospective adult spinal PA series published to date showed that patients who received GTR or STR tended to have a better OS compared to those who had only a biopsy (130.7 months versus 77.7 months, *p* = 0.274) [54]. At the time of recurrence, re-surgery should be considered. If re-surgery is not possible, treatment options include RT, chemotherapy, and targeted therapies that are playing an increasingly important role in these tumors.

An issue regarding the role of RT in recurrent spinal PA is that its efficacy remains debated [51,55]. Indeed, the use of adjuvant RT after surgery has been associated with a lower PFS in some retrospective series [45,56,57] and with a worse OS when compared to observation in another retrospective series [54]. Similarly, there is little data concerning the role of chemotherapy in spinal PA. In a retrospective series on 31 adult patients with spinal PA, the use of chemotherapy was associated with a worse outcome [45]. This finding likely reflects a selection bias, and there are several reports of patients with recurrent spinal PA in whom TMZ [58,59], carboplatin, vincristine, or bevacizumab resulted in prolonged disease control [50,60]. Finally, patients with BRAF-altered spinal PA can benefit from BRAF inhibitors. There is a clinical report of an adult patient with a spinal BRAF V600E PA treated with combined BRAF/MEK tyrosine kinase inhibitors in whom the treatment resulted in a one-year disease stabilization [59]. Importantly, new-generation BRAF inhibitors have demonstrated efficacy in PA with the BRAF–KIAA1549 fusion in recent clinical trials and may constitute in the future a promising treatment option for recurrent or unresectable spinal PA with a BRAF fusion [49,61]. Despite, limited data regarding the role of anti-BRAF-targeted therapies in spinal pilocytic astrocytomas, based on available data in their intracranial counterparts, there is a trend to consider anti-BRAF-targeted therapies as the treatment of choice in cases not amenable to resurgery. 

### 3.2. Spinal Diffuse Grade 2 Astrocytomas

Primary spinal diffuse grade 2 astrocytoma is an infiltrative tumor that tends to progress despite local treatment. Due to its low frequency and the recent change of tumor classification in 2021, its incidence is not well known. Unlike intracranial diffuse astrocytomas, intramedullary diffuse astrocytomas are rarely IDH-mutated [52,62,63] and can harbor BRAF mutations [52].

Surgical management of spinal astrocytoma is difficult, since these tumors are infiltrative, and therefore, GTR is difficult [64]. Nevertheless, retrospective studies have shown that the extent of surgical resection had a positive impact on survival [65,66]. If GTR or near GTR is achieved, observation is generally recommended although there are no prospective studies to support this recommendation [67]. When GTR is not possible, most experts recommend to consider adjuvant therapy. The role of postoperative RT and chemotherapy, however, remains controversial in the absence of prospective studies. In patients with incompletely resected spinal astrocytomas, postoperative RT has been retrospectively associated with a lower PFS (5-year PFS of 55.1% without RT vs. 22.9%) and OS (5-year OS of 79.5 months vs. 51.5%) [45,65,66,68]. However, these findings may reflect a selection bias, with patients who were treated with RT representing patients with more aggressive tumors. Several retrospective series assessed the role of different chemotherapy regimens used for intracranial gliomas in spinal astrocytomas at recurrence after RT [10,35,39,45,67,69,70,71]. Several studies showed that TMZ could have some effect on low-grade spinal astrocytoma [39,67,69,71]. For example, Chamberlain et al. reported a series that included both grade 2 and grade 3 astrocytomas (*n* = 22), in which 73% of patients showed a PR or SD as the best response, with a median PFS and OS of 14.5 and 23 months, respectively [67]. Other chemotherapy regimens that have been shown to result in potential disease control at recurrence include PCV (procarbazine–CCNU–vincristine) [72] and bevacizumab [35]. 

### 3.3. Spinal H3K27M-Altered Diffuse Midline Gliomas

Diffuse midline gliomas (DMGs) with H3K27 alterations (H3K27M-DMGs) are an aggressive type of glioma classified as grade 4 in the 2021 WHO classification [11]. These gliomas are characterized by loss of the histone H3K27 trimethylation mark, which is caused by the presence of K27M mutations in histone H3–3A, H3C14, or H3C2, or, more rarely, by overexpression of the EZHIP protein. A subset of H3K27M DMGs is also associated with recurrent EGFR alterations. Approximately 10% to 30% of H3K27M-DMG is located in the spinal cord [73,74,75], and H3K27M-DMG accounts for 40% to 60% of spinal cord gliomas, which histologically resemble high-grade astrocytomas [8,52,64,76,77]. Spinal cord H3K27M DMG has a poor prognosis, with a median OS of approximately 6 to 16 months. Treatment relies on maximal safe surgical resection followed by RT frequently associated with TMZ, although its role is unclear since most H3K27M-DMG is MGMT unmethylated [78]. At recurrence, the analysis of 50 patients included in five clinical trials has shown that 20% of H3K27M-DMG responded to ONC201, a selective dopamine receptor D2 antagonist [79,80]. However, H3K2M-DMG with a spinal location was excluded from this study due to the difficulty of evaluating radiological tumor response in spinal gliomas [80]. Two clinical trials are currently evaluating the role of first-line ONC201 in addition to RT in H3K27M-DMG. Importantly, these trials will include spinal H3K27M-DMG (Biomede 2 [NCT05476939] and Action trial [NCT05580562]). Finally, a subset of H3K27M-DMG including some cases with a spinal location harbor targetable alterations MAPK pathway alterations. These tumors seem to correspond to a distinct subtype of H3K27M-DMG associated with a better prognosis [81,82].

### 3.4. Spinal Glioblastomas and High-Grade Astrocytomas

Spinal glioblastomas (GBMs) and high-grade astrocytomas are a very rare entity, accounting for <1.5% of all spinal tumors [83,84]. The age at diagnosis is around 30–50 years [83,84,85]. The median OS of patients with spinal GBM is approximately 10–16 months and is generally considered as inferior to that of cerebral GBM [84]. The cervical–thoracic segment is the most common location, and cervical location has been related to a worse prognosis [83].

The management of spinal GBM is poorly defined because of its rarity. In addition, it is likely that many cases reported as spinal GBMs actually corresponded to spinal DMG-H3K27M. GTR is mostly impossible in this type of tumor and most cases undergo STR or biopsy [71,83,84,86]. In a large retrospective series on primary spinal GBM, which included 33 patients, GTR was associated with a higher rate of surgical morbidity but with no survival benefit compared to STR or biopsy [83]. In this series, postoperative treatment, whatever the type (i.e., TMZ alone, RT alone, or RT + TMZ), was associated with an improved outcome compared to patients who had only surgery. Median OS was 10.5 months, 11 months, and 16 months in patients treated with TMZ alone, RT alone, and RT + TMZ, respectively, compared to 3.4 months in those treated with surgery alone [83]. Based on this study and others, postoperative treatment of spinal GBM relies on RT + TMZ as in supratentorial GBM [5,20,45,71,83,84]. At recurrence, bevacizumab can improve symptoms and be associated with temporary disease control but its impact on OS is unclear [71,83,87].

### 3.5. Spinal High-Grade Astrocytomas with Piloid Features

High-grade astrocytoma with piloid features (HGAP) is a rare and recently described entity defined by a specific DNA methylation profile [88]. Around 10% of cases occur in NF1 patients and HGAP typically exhibits MAPK pathway alterations (e.g., NF1, BRAF, or FGFR1 mutations) in combination with CDKN2A/B deletion and/or mutations, loss of ATRX, and may have a methylated MGMT promoter [88,89]. Median age at diagnosis is around 40 years [48]. The 5-year OS rate for HGAP is approximately 50% [90]. HGAP is most frequently located in the posterior fossa but 10% to 15% affects the spine. Given its rarity and recent identification, the optimal management is unknown. After maximal safe surgical resection, it seems reasonable to treat HGAP patients with postoperative RT + TMZ. Given their frequent MAPK pathway alteration, HGAP could benefit from BRAF, MEK, or FGFR inhibitors [88], yet data are currently lacking to determine in which way HGAP can benefit from these treatments.

In conclusion, as for ependymomas, there is an important need for prospective studies in spinal astrocytomas to clarify the role of radiotherapy, chemotherapy, and targeted therapies in these tumors.

## 4. Spinal Glioneuronal Tumors

Spinal glioneuronal tumors are very rare and most frequently consist of gangliogliomas and diffuse leptomeningeal glioneuronal tumors (DLGNTs).

### 4.1. Spinal Gangliogliomas

Gangliogliomas are defined as WHO grade 1 tumors by the 2021 WHO Classification [11,47]. They most frequently affect the brain, especially the temporal lobe but can rarely be located in the spine. Gangliogliomas are characterized by MAPK pathway alterations, especially BRAF V600E mutations that occur in about two-thirds of cases [47,91,92,93,94,95]. BRAF mutations are associated with a worse prognosis but represent an interesting therapeutic target [96]. The treatment of spinal gangliogliomas relies on surgical resection. Based on recommendations regarding their intracranial counterpart, a watch-and-wait approach is recommended for patients after complete or subtotal resection. At recurrence, if re-surgery is not possible and if a MAPK pathway alteration is present, a targeted therapy is an interesting therapeutic option [94,96,97,98]. Garnier et al. reported the case of a patient with a recurrent cervical ganglioglioma BRAFV600E who achieved a long-lasting response to vemurafenib [91]. In the absence of the MAPK pathway targetable alteration, RT and/or chemotherapy (e.g., TMZ or carboplatin) can be considered although data are very limited regarding the efficacy of these treatments [91].

### 4.2. Spinal DLGNT

Diffuse leptomeningeal glioneuronal tumor (DLGNT) is a rare CNS neoplasm usually diagnosed in pediatric or young adult patients, characterized by widespread leptomeningeal growth and oligodendroglial-like cytology. However, they can sometimes present as a solitary spinal cord mass without obvious leptomeningeal involvement [99,100,101]. DLGNTs are characterized by a high rate of KIAA1549–BRAF gene fusion (the most frequent molecular alteration in DLGNT) associated with either chromosome 1p deletion or 1p/19q codeletion, in the absence of IDH mutations [102,103]. Previous studies have shown that a Ki-67 > 7% is associated with poor prognosis when it is present, and it is the most important prognostic factor in DLGNT survival [103]. In addition, two subgroups of DLGNT (MC-1 and MC-2) can be distinguished based on methylation profiling, with one subgroup (MC-1) being associated with a better outcome [104]. Based on retrospective studies, DLGNT management relies on chemotherapy, craniospinal RT, and sometimes surgery in case of solitary mass presentation [99,100]. Chemotherapy regimens that can result in disease control include the association of carboplatin and vincristine, PCV, and TMZ [105,106,107]. There is preliminary evidence that DLGNT could benefit from anti-MAPK pathway targeted therapies. Responses to vemurafenib (anti-BRAF treatment) and trametinib (MEK inhibitor) have been reported [108]. In the future, new-generation RAF inhibitors such as tovorafenib could constitute an interesting therapeutic option in DLGNT with a BRAF fusion [107].

## 5. Spinal Hemangioblastomas

Hemangioblastomas represent 2–6% of intramedullary tumors [1,5]. These are benign, highly vascularized lesions primarily located on the cervical spine. They express a high amount of VEGF, which drives angiogenesis, a process that explains their highly vascular nature [109]. Although the majority of cases are sporadic [110], 15–40% of hemangioblastomas occur as a manifestation of von Hippel–Lindau (VHL) disease [10]. Approximately 60–80% of VHL patients have hemangioblastomas [111,112], which are frequently multiple [110]. Patients with spinal hemangioblastomas should be screened for other manifestations of VHL (with an MRI of the brain (cranial hemangioblastoma), neuro-ophthalmologic examination (retinal hemangioblastoma), and CT imaging of the body (renal cell carcinomas and pheochromocytoma)) [10,113].

The standard treatment for large, symptomatic hemangioblastomas is GTR [114]. In cases in which surgery is considered as not possible or too risky, several retrospective studies have shown that stereotactic radiosurgery (SRS) or fractionated external beam RT was an alternative to surgery resulting in a local control rate of 60% to 90% [111,115,116]. The target for SRS is typically the contrast-enhancing tumor without margin, and prescription doses from 12 to 20 Gy have successfully been used [116]. In patients with disseminated or progressive disease after surgery, SRS or RT is the most frequently used treatment for disease control [10].

In cases of multiple or non-resectable VHL-related hemangioblastomas, systemic treatment is another option [116]. VEGF and VEGF receptors are expressed in hemangioblastoma; therefore, anti-angiogenic therapy has been tested as salvage therapy in CNS hemangioblastomas [114,116]. Several anti-angiogenic treatments such as multi-target tyrosine kinase inhibitors (semaxanib, sunitinib, vatalanib, pegaptanib, vandetanib, dovitinib) [116,117,118], thalidomide [119], the VEGF-targeting antibodies ranibizumab and bevacizumab, and interferon alfa-2a have been retrospectively shown to enable disease control in hemangioblastomas but the most promising treatment is belzutifan [116,118]. Belzutifan is a hypoxia-inducible factor-2 alpha (HIF-2α) inhibitor approved in the US for the treatment of renal cancer, pancreatic neuroendocrine tumors, and hemangioblastomas in VHL patients [120]. In a prospective trial, belzutifan has been shown to elicit responses in around 30% of VHL-related hemangioblastoma with a 30% response rate and ~30–50% reduction in their sizes over the course of treatment [120]. This treatment is an interesting therapeutic option in progressive VHL disease-related hemangioblastomas that are no longer candidates for surgery [118].

## 6. Conclusions

Intramedullary spinal cord tumors represent a heterogeneous group of rare tumors. Within recent years, important progress has been made in their classification and molecular characterization, and promising actionable alterations have been identified in several subgroups. Nevertheless, the role of RT, chemotherapy, and targeted therapies remains poorly defined. International collaborative efforts will be necessary to launch prospective studies to allow for defining the optimal management of intramedullary spinal cord tumors. Meanwhile, patients should be treated as much as possible within clinical trials and/or be registered in dedicated registries, and cases should be discussed within specialized tumor boards [121].

## Figures and Tables

**Table 1:** Tumor classification.

Tumor Diagnosis	Genetic Alterations
**Ependymal tumors**	
Spinal subependymomas (grade 1)	Unknown
Spinal ependymomas (grade 2)	Chromosome 22 deletion (1 copy loss) NF2 mutation or deletion
Spinal ependymomas (grade 3)	Chromosome 22 deletion (1 copy loss) NF2 mutation or deletion
Spinal ependymomas MYCN (grade 3)	MYCN amplification, specific methylation profile
Myxopapillary ependymomas (grade 2)	Unknown
**Spinal astrocytoma**	
Pilocytic astrocytoma (PA)	MAPK pathway alterations, especially BRAF V600E mutation, KIAA1549–BRAF fusion
Diffuse astrocytoma (DA)	IDH1/2 mutation (rarely), BRAF V600E mutation, ATRX mutation, TP53 mutation
Spinal glioblastoma IDH wildtype (GBM)	EGFR amplification, PTEN homozygous deletion, 7p gain/10 homozygous deletion, TERT promoter mutation, TP53
Diffuse midline glioma H3 K27M altered (DMG-H3)	H3 K27M mutation, EGFR alteration, MAPK alterations, TP53 mutation, ATRX mutation
High-grade astrocytoma with piloid features (HGAP)	NF1 mutation, MAPK alterations, CDKN2A/B deletion and/or mutations, loss of ATRX, MGMT promoter methylation
**Spinal glioneuronal tumor**	
Gangliogliomas	BRAF V600E mutation or other MAPK pathway alteration
Diffuse leptomeningeal glioneuronal tumors (DLGNT)	KIAA1549–BRAF fusion, 1p/19q codeletion, IDHwt
**Spinal hemangioblastoma**	VHL gene mutation

ATRX, alpha-thalassemia/mental retardation X-linked; CDKN2A/B, cyclin-dependent kinase inhibitor 2A/B; IDH, isocitrate dehydrogenase; MAPKs, mitogen-activated protein kinases; NF, neurofibromatosis; MGMT, methylguanine-DNA methyltransferase; TP53, tumor protein 53; VHL, Von Hippel–Lindau; wt, wild type.

**Table 2:** Summary regarding the role of radiotherapy, chemotherapy, and targeted therapies.

Tumor	Radiotherapy	Chemotherapy	Targeted Therapy
**Ependymal tumors**			
Spinal subependymomas (grade 1)	Not recommended	Not recommended	Not known
Spinal ependymomas (grade 2)	Focal RT (45 to 54 Gy) if GTR is not possible or at recurrence	At recurrence if no local treatment is possible: TMZ or TMZ + lapatinib or platin-based regimens or etoposide or bevacizumab-based regimens	Not known
Spinal ependymomas (grade 3) Spinal ependymomas MYCN (grade 3)	Focal RT (45 to 54 Gy) after GTR or STR CSI (<36 Gy at 1.5–1.8 Gy/fr) if leptomeningeal dissemination + boost 45–54 Gy		
Myxopapillary ependymomas (grade 2)	Focal RT (45 to 54 Gy) if GTR is not possible or at recurrence	If progression/non-resectable: TMZ or TMZ + olaparib	Not known
**Spinal astrocytoma** Pilocytic astrocytoma	Therapeutic option at progression	If progression/non-resectable: TMZ or carboplatin or vincristine or bevacizumab	BRAF and or MEK inhibitors
Diffuse astrocytoma	Focal RT (45 to 54 Gy) if GTR is not possible or at recurrence	If progression/non-resectable: TMZ or PCV (procarbazine–CCNU–vincristine) or bevacizumab	Not known
Spinal glioblastoma IDH wildtype	Focal RT ± CT after surgery	TMZ If progression: lomustine, bevacizumab	Not known
Diffuse midline glioma H3 K27M altered	Focal RT ± C	Lomustine, bevacizumab	ONC201
High-grade astrocytoma with piloid features	Focal RT ± CT after surgery	TMZ	BRAF, MEK, or FGFR inhibitors
**Spinal glioneuronal tumor**			
Gangliogliomas	No clear recommendations	No clear recommendations TMZ or carboplatin	BRAF/MEK tyrosine kinase inhibitors
Diffuse leptomeningeal glioneuronal tumors	CSI	Carboplatin and vincristine or PCV or TMZ	BRAF/MEK tyrosine kinase inhibitors
**Spinal hemangioblastoma**	SRS (12 to 20 Gy) or fractionated RT if surgery is not possible		If multiple/not surgical VHL-related: Belzutifan, tyrosine kinase inhibitors, bevacizumab

CT, chemotherapy; CSI, craniospinal irradiation; GTR, gross total resection; Gy, grays; IDH, isocitrate dehydrogenase; PCV, procarbacine–CCNU–velustine; RT, radiotherapy; STR, subtotal resection; SRS, stereotactic radiosurgery; TMZ, temozolomide.

## Data Availability

No new data were created or analyzed in this study. Data sharing is not applicable to this article.
